# Association of the abdominal aortic calcification with all-cause and cardiovascular disease-specific mortality: Prospective cohort study

**DOI:** 10.1371/journal.pone.0314776

**Published:** 2025-01-16

**Authors:** Chang Sheng, Zhou Cai, Pu Yang

**Affiliations:** 1 Department of Vascular Surgery, Xiangya Hospital, Central South University, Changsha, Hunan, China; 2 National Clinical Research Center for Geriatric Disorders, Xiangya Hospital, Central South University, Changsha, Hunan, China; National University of Singapore, SINGAPORE

## Abstract

**Background:**

Abdominal aortic calcification (AAC) is a prevalent form of vascular calcification associated with adverse cardiovascular outcomes. While previous studies on AAC and cardiovascular risk exist, many have limitations such as small sample sizes and limited clinical significance outcomes. This study aims to prospectively investigate the association between AAC and all-cause and cardiovascular disease (CVD)-specific mortality rates in a nationally representative sample of adults in the United States, using data from the National Health and Nutrition Examination Survey (NHANES).

**Methods:**

The study, conducted on NHANES participants aged 40 years or older during the 2013–2014 cycle, assessed AAC using the Kauppila scoring system. Demographic characteristics, mortality data, and comorbid factors such as age, gender, diabetes, and hypertension were considered. Statistical analyses, including weighted percentages, Kaplan-Meier survival curves, and multivariable Cox proportional hazards regression models, were employed to evaluate the associations between AAC and mortality risks.

**Results:**

After analyzing a final sample of 2717 participants, the study found a significant association between severe AAC (SAAC) and higher all-cause mortality risk (HR 1.70, 95% CI 1.17–2.48). The dose-response relationship indicated an increased risk with higher AAC scores. However, no independent association was observed between AAC and cardiovascular mortality. Stratified analysis revealed variations in the AAC-all-cause mortality association based on gender and hypertension.

**Conclusion:**

This population-based study provides valuable insights into the prospective association between AAC and all-cause mortality, emphasizing the potential role of AAC assessment in identifying individuals at higher risk.

## Introduction

Abdominal aortic calcification (AAC), a prevalent form of vascular calcification [1], is commonly observed in the general population, and its incidence and severity increase with advancing age [2]. Numerous epidemiological studies have established an association between AAC and adverse cardiovascular outcomes, including stroke [3], coronary heart disease [4], and myocardial infarction [5]. The escalation in the severity of aortic calcification has been recognized as a predictor of specific cardiovascular events and overall mortality [6–10]. Some reports suggest that the visible amount of AAC in imaging tests determines the risk of cardiovascular events, fatal cardiovascular events, and all-cause mortality, with the highest risk observed in patients with advanced calcification [11–13].

Despite the recognized importance of AAC, existing studies suffer from limitations such as relatively small sample sizes, limited reporting of clinically significant outcomes, and a need to establish the relevance of AAC in various patient subgroups. The National Health and Nutrition Examination Survey (NHANES) is a periodic, cross-sectional health survey program that utilizes a stratified, multistage, and probability-cluster design to obtain a nationally representative sample of non-institutionalized individuals in the United States. Combining interviews and medical examinations, NHANES collects a wide range of demographic, socioeconomic, dietary, physiological, and laboratory information, providing a robust platform to investigate the prognostic implications of AAC.

To contribute to the existing evidence, our study prospectively explores the relationship between AAC and all-cause, as well as cardiovascular mortality rates among adult individuals in the United States. Furthermore, we also aim to determine the strength of this association and assess whether it varies among populations with different comorbid factors such as gender, age, hypertension, and diabetes.

## Methods

### Study design and participants

Administered by the National Center for Health Statistics (NCHS), NHANES is conducted with approval from the institutional ethics review board of NCHS, and written informed consent is obtained from all participants. The research data were obtained on February 9, 2024, ensuring that individual participants remained anonymous throughout the study. Our study specifically focused on NHANES participants aged 40 years or older during the 2013–2014 cycle. Inclusion criteria encompassed individuals with complete survival information, AAC measurements, and relevant demographic variables. At the outset, our initial study cohort consisted of 10175 participants. Subsequently, we refined the sample by excluding individuals below the age of 40 years (n = 6360), those with incomplete AAC data (n = 675), insufficient survival data (n = 9), and participants with missing covariate information (n = 690). A comparison of all-cause mortality between the 690 participants with missing covariate data and those with complete data revealed no significant difference. Consequently, we derived a final analytical sample comprising 2717 participants.

The analyzed cohort exhibited common characteristics of younger age, higher PIR, cohabitation status, and lower educational levels. Moreover, the study cohort predominantly consisted of non-Hispanic white participants (S1 Table). It should be noted that the differences between the included and excluded subsets highlight the need for cautious extrapolation of the study results to a broader population.

### Study variables

#### Demographic characteristics

Demographic Characteristics: Information obtained through questionnaires during in-home interviews, categorized age into two groups (40–59 years or ≥ 60 years). Race included non-Hispanic White, non-Hispanic Asian, Mexican American, Other Hispanic, non-Hispanic Black, and Other Race. The PIR evaluated income in relation to federal poverty thresholds and was divided into three categories: < 1.38 (indicating low income), 1.38–3.99 (representing middle income), and ≥ 4.00 (reflecting high income) [14]. Marital status was characterized as either married/living with a partner or single [15]. Educational levels were categorized into college graduate or above, some college or associate’s degree, and high school degree/equivalency or less [15].

#### Definition of mortality

Mortality Definition: Baseline information from NHANES 2013–2014 was linked with mortality records sourced from the National Death Index death certificates, extending through December 31, 2019. The linkage employed a probabilistic matching algorithm to ascertain mortality status. The study’s outcomes encompassed both all-cause mortality and mortality specific to cardiovascular disease (CVD) (coded I00–I09, I11, I13, I20–I51, and I60–I69), utilizing the International Classification of Diseases, Tenth Revision.

#### Measurements and definition of AAC

AAC Measurements and Definition: The degree of AAC was evaluated using the Kauppila scoring system [16], ranging from 0 to 3 for each of the eight segments, with a total score of 24. A widely accepted threshold designating severe abdominal aortic calcification (SAAC) was applied when the AAC score exceeded 6. In contrast, mild–moderate AAC (MAAC) was defined as a score ranging from 1 to 6 points [15, 17].

### Statistical methods

Considering the complex sampling design of NHANES, all analyses were conducted by incorporating sample weights, clustering, and stratification to ensure nationally representative estimates. Weighted percentages presented categorical variables, and weighted means were used for continuous variables.

The decision to categorize AAC was motivated by the evident skewness in the data, with around thirty percent of participants reporting AAC. Kaplan-Meier survival curves were employed to compute cumulative mortality, utilizing three score categories of AAC metrics (no, mild–moderate, severe). Survey-weighted multivariable Cox proportional hazards regression models were then utilized to derive hazard ratios (HRs) and corresponding 95% confidence intervals (CIs) to assess the associations of AAC with the risks of all-cause and CVD-specific mortality. Model 1 did not incorporate adjustments for any covariates; Model 2 was adjusted for gender, age (as a continuous variable), race, education levels, marital status, and poverty ratio (as a continuous variable); Model 3 expanded on Model 2 by incorporating hypertension and diabetes. Schoenfeld residuals were used to test the proportional hazards assumption, and no violation was observed. To visualize the dose-response association of AAC levels with all-cause and CVD-specific mortality, we employed the restricted cubic spline (RCS) model without weights. This choice was made due to the unavailability of an RCS model specifically designed for complex, multistage sampling survey data.

To probe demographic-related disparities within susceptible subpopulations, we conducted stratified analyses based on age strata, sex, poverty ratio, hypertension, and diabetes. The significance of interactions was assessed by determining the *P* values for the product terms between AAC and the stratified factors. All statistical analyses were performed using R software (version 4.2.1), considering a two-sided P value of less than 0.05 as statistically significant.

## Results

### Participants characteristics

Following the application of weights, the study encompassed a total of 111799277 participants. Table 1 provides a summary of the baseline characteristics of the study population, organized by AAC level. The weighted mean age of the study participants was 57.42±11.53 years, with the weighted proportion of females being 51.34%. Statistically significant differences (all *P* values < 0.05) were observed in age, poverty status, education level, marital status, smoking, albuminuria, chronic kidney disease, hypertension, diabetes, CVD, albumin, and across different AAC levels (Table 1). Specifically, participants with SAAC were more likely to be older, economically disadvantaged, single, and smokers. They were also more likely to have concomitant renal insufficiency, diabetes, and cardiovascular diseases, as well as lower educational levels.

**Table 1:** Baseline characteristics of the study population.

Characteristic	Overall N = 111799277	No AAC N = 79718967	MAAC N = 23402727	SAAC N = 8677583	*P* value
Age, years	57.42 (11.53)	54.93 (10.42)	60.87 (11.55)	71.01 (9.12)	<0.001
Age strata, %					<0.001
40–59	66701183 (59.66%)	54134866 (67.91%)	11444718 (48.90%)	1121600 (12.93%)	
60+	45098094 (40.34%)	25584101 (32.09%)	11958009 (51.10%)	7555983 (87.07%)	
Sex, %					0.611
Male	54401504 (48.66%)	38810090 (48.68%)	11733521 (50.14%)	3857892 (44.46%)	
Female	57397773 (51.34%)	40908877 (51.32%)	11669206 (49.86%)	4819691 (55.54%)	
Race, %					0.107
Mexican American	7279573 (6.51%)	5784260 (7.26%)	1117231 (4.77%)	378083 (4.36%)	
Other Hispanic	4826861 (4.32%)	3686855 (4.62%)	940534 (4.02%)	199472 (2.30%)	
Non-Hispanic White	80879994 (72.34%)	56054541 (70.32%)	17826198 (76.17%)	6999257 (80.66%)	
Non-Hispanic Black	10770883 (9.63%)	8345197 (10.47%)	1918524 (8.20%)	507162 (5.84%)	
Non-Hispanic Asian	5625387 (5.03%)	4192816 (5.26%)	1118065 (4.78%)	314505 (3.62%)	
Other Race	2416577 (2.16%)	1655298 (2.08%)	482175 (2.06%)	279104 (3.22%)	
PIR	3.17 (1.63)	3.28 (1.63)	2.96 (1.62)	2.79 (1.52)	0.032
PIR strata, %					0.017
<1.38	22404818 (20.04%)	15259255 (19.14%)	5080976 (21.71%)	2064586 (23.79%)	
≥1.38 and <3.99	44455719 (39.76%)	29595626 (37.12%)	10572743 (45.18%)	4287351 (49.41%)	
≥3.99	44938740 (40.20%)	34864086 (43.73%)	7749008 (33.11%)	2325646 (26.80%)	
Education level, %					<0.001
High school degree/equivalency or less	40606804 (36.32%)	26897504 (33.74%)	9576458 (40.92%)	4132842 (47.63%)	
Some college or associates degree	34010179 (30.42%)	23970454 (30.07%)	7419660 (31.70%)	2620065 (30.19%)	
College Graduate or above	37182294 (33.26%)	28851009 (36.19%)	6406609 (27.38%)	1924676 (22.18%)	
Marital status, %					<0.001
Married/Living with partner	76338201 (68.28%)	56430650 (70.79%)	15566583 (66.52%)	4340968 (50.03%)	
Single	35461076 (31.72%)	23288317 (29.21%)	7836144 (33.48%)	4336615 (49.97%)	
BMI, kg/m^2^					0.766
<25.0	29903589 (26.75%)	21063261 (26.42%)	6374560 (27.24%)	2465768 (28.42%)	
≥25.0	81895688 (73.25%)	58655706 (73.58%)	17028167 (72.76%)	6211815 (71.58%)	
Smoking, %					0.001
Never	59888254 (53.57%)	45802771 (57.46%)	10829399 (46.27%)	3256084 (37.52%)	
Former	31649422 (28.31%)	20437866 (25.64%)	7424490 (31.72%)	3787065 (43.64%)	
Now	20261601 (18.12%)	13478330 (16.91%)	5148838 (22.00%)	1634434 (18.84%)	
Albuminuria, %	12082469 (10.81%)	7628036 (9.57%)	2796959 (11.95%)	1657474 (19.10%)	0.002
CKD group, %					<0.001
No CKD	90206060 (80.69%)	67412133 (84.56%)	18170427 (77.65%)	4623499 (53.28%)	
Stages 1–2	8751992 (7.83%)	6012082 (7.54%)	1783931 (7.62%)	955978 (11.02%)	
Stages 3	12154239 (10.87%)	5859156 (7.35%)	3291125 (14.06%)	3003958 (34.62%)	
Stages 4–5	686986 (0.61%)	435596 (0.55%)	157244 (0.67%)	94147 (1.08%)	
Total cholesterol/HDL	3.88 (1.38)	3.85 (1.40)	4.00 (1.40)	3.77 (1.17)	0.124
Diabetes, %	20752507 (18.56%)	12453375 (15.62%)	4985826 (21.30%)	3313307 (38.18%)	<0.001
Hypertension, %	49958133 (44.69%)	30899192 (38.76%)	12790754 (54.65%)	6268187 (72.23%)	<0.001
CVD, %	11213750 (10.03%)	5673310 (7.12%)	3500128 (14.96%)	2040312 (23.51%)	<0.001
Albumin, g/dL	4.25 (0.30)	4.26 (0.30)	4.25 (0.32)	4.20 (0.27)	0.036
Serum total calcium, mg/dL	9.46 (0.36)	9.44 (0.36)	9.49 (0.35)	9.49 (0.35)	0.205
Serum phosphorus, mg/dL	3.79 (0.56)	3.79 (0.56)	3.77 (0.54)	3.89 (0.57)	0.170
Total 25-hydroxyvitamin D, nmol/L	74.85 (29.31)	74.16 (28.99)	74.37 (29.51)	82.49 (30.66)	0.001

Data are presented as mean (SD), n (%), and *P* value.

Analysis conducted: Wilcoxon rank-sum test for complex survey samples; chi-squared test with Rao & Scott’s second-order correction.

Abbreviation: PIR = poverty income ratio, BMI = body mass index, CKD = chronic kidney disease, CVD = cardiovascular disease, AAC = abdominal aortic calcification, SAAC = severe AAC, MAAC = mild–moderate AAC.

**Fig 1:**
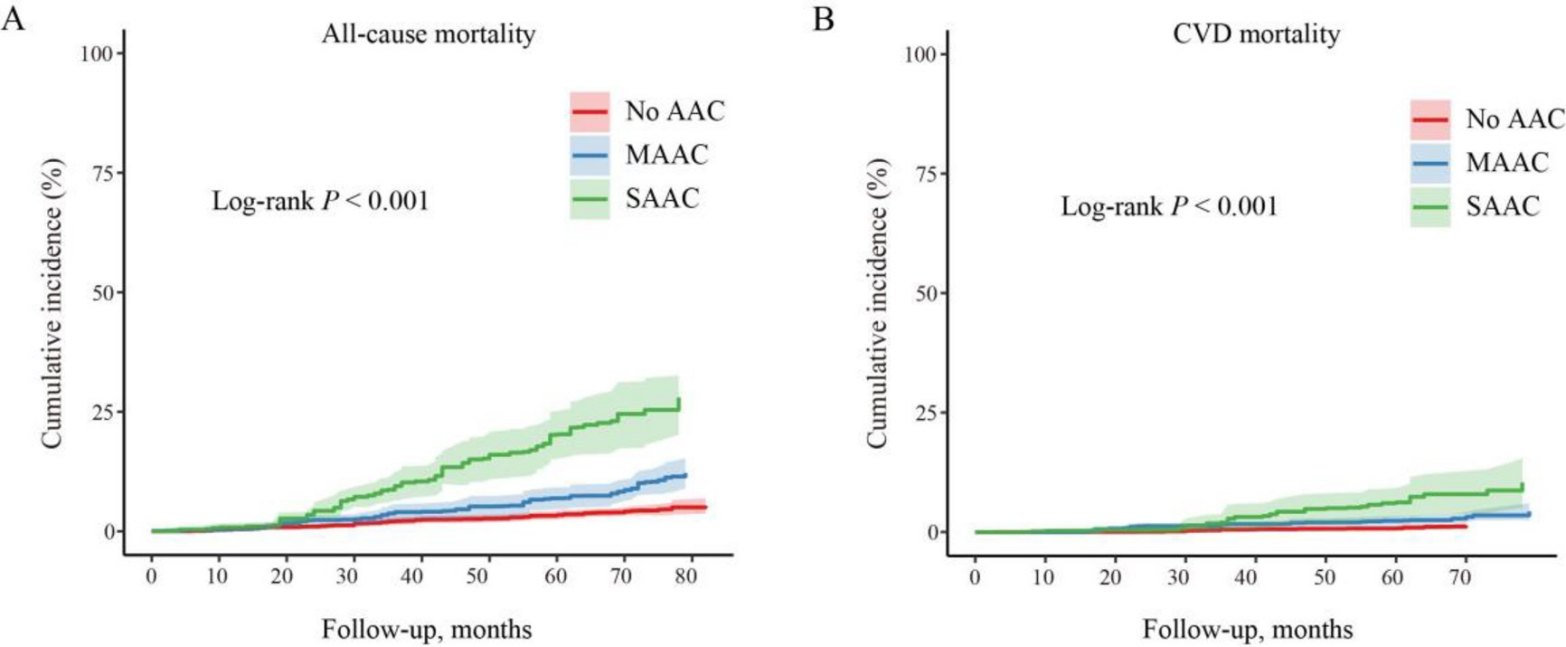
Kaplan–Meier curves for cumulative all-cause and CVD-specific mortality by AAC level. Abbreviations: CVD = cardiovascular disease, AAC = abdominal aortic calcification, SAAC = severe AAC, MAAC = mild–moderate AAC.

Over a median follow-up period of 72 months (up to 85 months), there were 235 all-cause deaths, including 78 attributed to CVD. The weighted all-cause mortality rates were 5.3%, 12.2%, and 26.6% for the no, mild–moderate, and severe AAC groups, respectively. Similarly, the weighted CVD-specific mortality rates were 1.3%, 4.3%, and 10.4% for the respective AAC groups., respectively. Participants with a higher AAC score exhibited a significantly elevated cumulative incidence rate of both all-cause and CVD-specific mortality (*P* < 0.001 for all log-rank tests, Fig 1).

### Survival analysis

**Fig 2:**
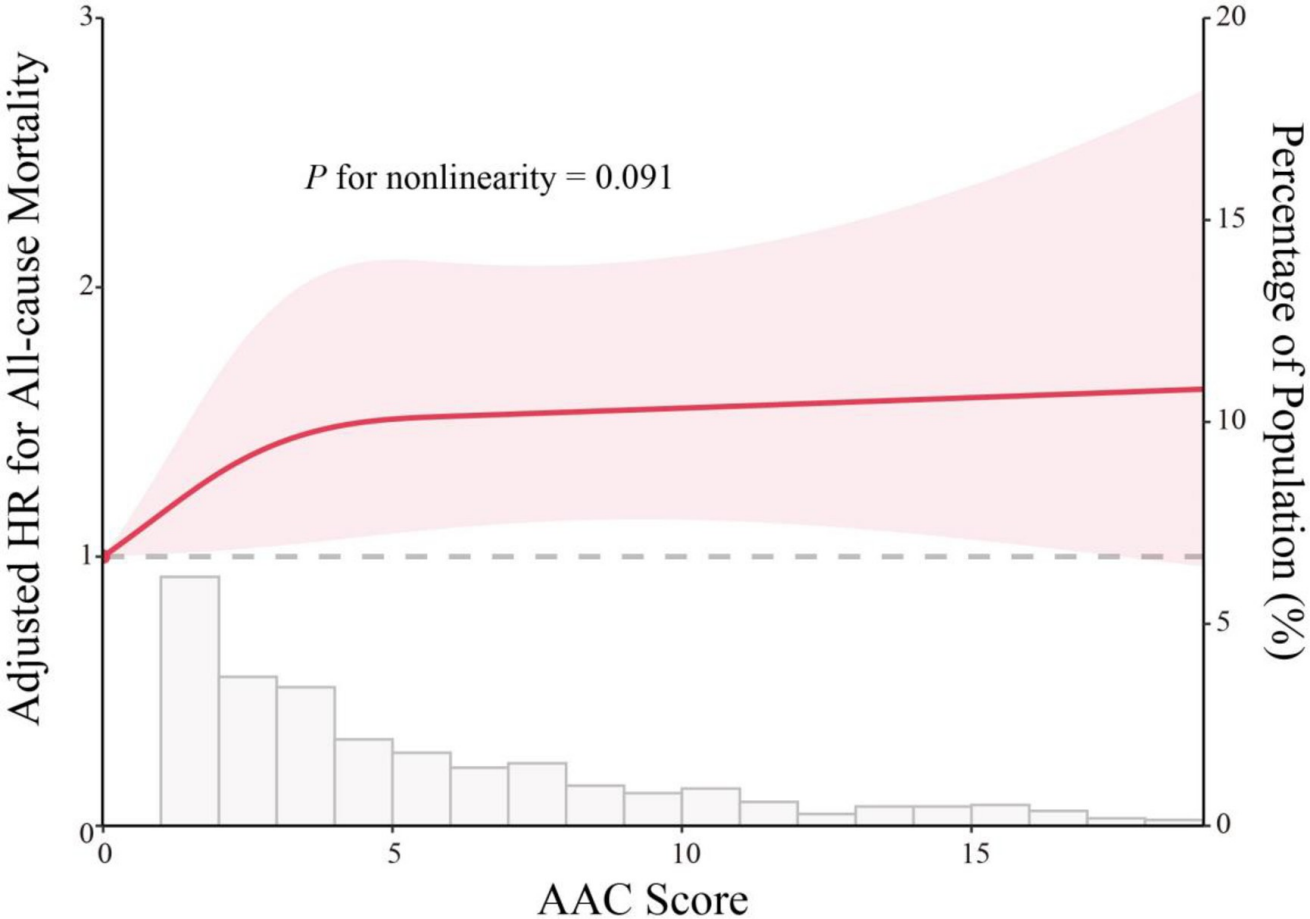
The restricted spline curve depicts the association between AAC score and all-cause mortality. The red line and shaded area represent the HR and 95% CI, respectively. The histogram illustrates the score distribution within the population. The HR (95% CI) was adjusted using Model 3, considering sex, age, race, marital status, education, poverty level, hypertension, and diabetes. Abbreviation: AAC = abdominal aortic calcification, HR = hazard ratio, CI = confidence interval.

In the fully adjusted model (multivariable model 3), individuals with SAAC exhibited a higher risk of all-cause mortality in comparison to participants with no AAC (hazard ratio [HR] 1.70, 95% confidence interval [CI] 1.17–2.48). The multivariate-adjusted HR for each one-point increase in AAC score associated with all-cause mortality was 1.04 (95% CI 1.02–1.07; refer to Table 2). Notably, there were linear dose–response associations of AAC score with all-cause mortality (*P* for non-linearity >0.05; see Fig 2), suggesting that the risk of all-cause mortality increased linearly as the AAC score increased.

**Table 2:** Survey-weighted association of AAC with all-cause and CVD-specific mortality.

	Model 1		Model 2		Model 3	
Characteristic	HR (95% CI), *P* Value	*P* for trend	HR (95% CI), *P* Value	*P* for trend	HR (95% CI), *P* Value	*P* for trend
All-cause mortality						
No AAC	Reference		Reference		Reference	
MAAC	2.29 (1.64, 3.19), < 0.001		1.34 (0.96, 1.88), 0.085		1.31 (0.95, 1.82), 0.103	
SAAC	6.31 (4.90, 8.14), < 0.001	< 0.001	1.85 (1.28, 2.67), < 0.001	0.003	1.70 (1.17, 2.48), 0.005	0.019
AAC score ^a^	1.15 (1.13, 1.16), < 0.001		1.05 (1.03, 1.07), < 0.001		1.04 (1.02, 1.07), 0.001	
CVD mortality						
No AAC	Reference		Reference		Reference	
MAAC	2.74 (1.63, 4.60), < 0.001		1.51 (0.88, 2.59), 0.135		1.50 (0.88, 2.56), 0.140	
SAAC	7.22 (4.00, 13.06), < 0.001	< 0.001	1.73 (0.92, 3.26), 0.092	0.210	1.58 (0.81, 3.09), 0.179	0.299
AAC score ^a^	1.16 (1.12, 1.19), < 0.001		1.05 (1.01, 1.09), 0.020		1.04 (1.00, 1.08), 0.071	

^a^: The analysis was performed per a 1-point increase in the continuous variable.

Data are presented as HR, 95% CI, and *P* value.

Model 1 was adjusted for none.

Model 2 was adjusted for sex, age, race, marital status, education, and poverty level.

Model 3 was adjusted for sex, age, race, marital status, education, poverty level, hypertension, and diabetes.

Abbreviation: HR = hazard ratio, CI = confidence interval, AAC = abdominal aortic calcification, SAAC = severe AAC, MAAC = mild–moderate AAC, CVD = cardiovascular disease.

After adjusting for potential confounders, neither MAAC (HR 1.50, 95% CI 0.88–2.56) nor SAAC (HR 1.58, 95% CI 0.81–3.09) was independently associated with CVD-specific mortality risk. Additionally, with each incremental point increase in AAC score, there was no significant rise in the risk of CVD mortality (HR 1.04, 95% CI 1.00–1.08).

### Stratified analysis

**Fig 3:**
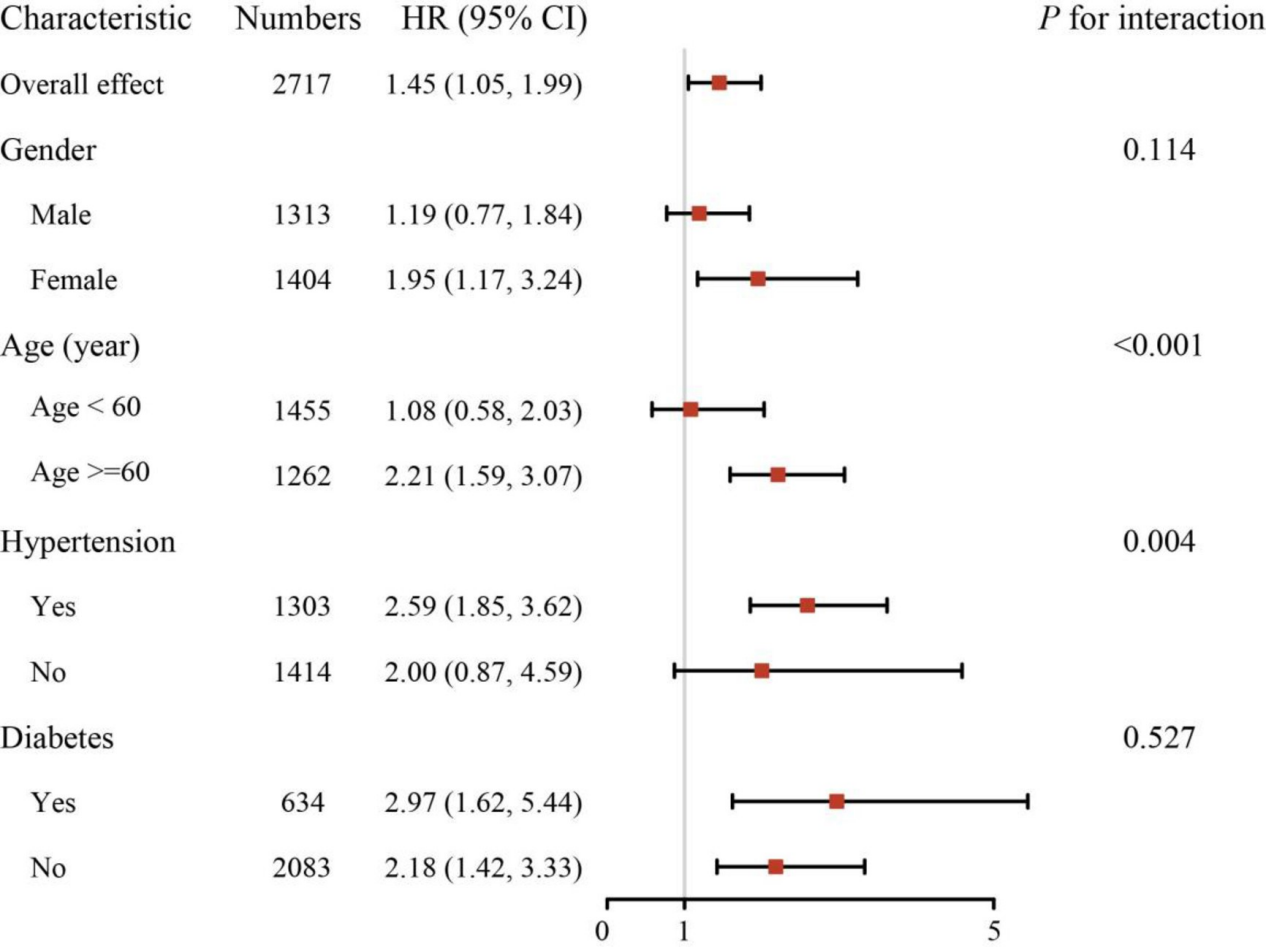
Subgroup analysis for the association between AAC and all-cause mortality. The HR (95% CI) was adjusted using Model 3, considering sex, age, race, marital status, education, poverty level, hypertension, and diabetes except the corresponding stratification variable. Abbreviation: HR = hazard ratio, CI = confidence interval.

To enhance the applicability of the study outcomes, this analysis segment divides AAC into two groups: the AAC group (AAC score ≥ 1 point) and the non-AAC group. The results of subgroup analyses are summarized in Fig 3. We observed that the AAC group had higher all-cause mortality than the non-AAC group. In most subgroups, there was a positive correlation between AAC and all-cause mortality. However, we noted that the relationship between AAC and all-cause mortality was influenced by the interactions of gender and hypertension (gender: *P* Value<0.001 for interaction; hypertension: *P* Value = 0.004 for interaction).

The direct association between AAC and all-cause mortality was not evident in males, participants younger than 60 years, and participants without hypertension. Conversely, the direct association between AAC and all-cause mortality was observed in females, participants aged 60 years or older, and those with hypertension. These findings underscore the importance of considering demographic and clinical factors in assessing the impact of AAC on all-cause mortality.

## Discussion

Utilizing a substantial representative sample of U.S. adults, our study demonstrates that individuals with elevated AAC levels face a heightened risk of all-cause mortality. Specifically, compared to patients without AAC, those with SAAC experience a 70% higher risk of all-cause mortality. Furthermore, for each additional point increase in AAC score, participants experience a 4% higher risk of all-cause mortality. However, the association with CVD-specific mortality was not as pronounced after adjusting for confounding factors. There is an approximately linear dose-response relationship between increases in AAC score and the elevated risk of all-cause mortality. Subgroup analyses revealed that the correlation between AAC and all-cause mortality remains significant in females, older individuals, and those with hypertension. Age and hypertension status were identified as important factors modifying the relationship between AAC and the risk of all-cause mortality. These results provide new evidence for the prognostic value of AAC, proving to be practical in predicting future rates of all-cause mortality. Incidentally discovered AAC in patients without known cardiovascular risk factors may necessitate further cardiovascular diagnostic testing.

Previous studies have primarily focused on specific populations such as dialysis patients and those with chronic kidney disease [18–20]. Our study targets the general population, expanding the breadth of knowledge in this area. There exists evidence supporting the promotive role of AAC in diabetes, cardiovascular diseases, and late-life mortality [2, 7, 9, 19]. A comprehensive systematic review and meta-analysis uncovered that individuals with advanced abdominal aortic calcification (AAC) face an elevated risk of cardiovascular events (risk ratio [RR] 1.83, 95% CI 1.40–2.39), all-cause mortality (RR 1.98, 95% CI 1.55–2.53), and fatal cardiovascular events (RR 1.85, 95% CI 1.44–2.39) [8]. Nevertheless, it is crucial to acknowledge that the encompassed studies were restricted to patients with chronic kidney disease and the elderly population, possibly amplifying the association between AAC and the risk of mortality. There are also conflicting study results, such as the research conducted by Ohya et al., which recruited 137 patients [21]. They reported that AAC is not a significant prognostic factor for all-cause mortality (HR 1.02, 95% CI 0.99–1.04). Of course, due to the limited sample size, the strength of evidence is insufficient. In alignment with earlier investigations, this study affirms that affordable and widely accessible imaging modalities can be employed to identify populations characterized by a notably heightened risk of mortality [9]. Our study’s linear dose-response findings suggest that any improvement is significant, particularly for patients with lower AAC scores, which is a novel result compared to previous research [8].

**Notably, our study contributes to the literature by using NHANES, offering a larger and more diverse sample, thus enhancing the generalizability of the results.** The stratified analysis revealed gender and hypertension as modifiers of the association between AAC and all-cause mortality. Hypertension is significantly associated with an elevated risk of CVD-specific and all-cause mortality [22]. Hypertension also mediates the relationship between aortic calcification and arterial stiffness, left ventricular hypertrophy, and diastolic dysfunction [23]. This emphasizes the importance of considering demographic factors in understanding the nuanced impact of AAC on mortality outcomes. Future research should explore the mechanisms underlying these variations and tailor preventive strategies accordingly. The prospective design enhances the credibility of the observed associations. However, limitations include the observational nature of the study, potential for residual confounding, and the exclusion of certain population segments, highlighting the need for cautious interpretation. **Additionally, it must be acknowledged that, during the analysis, due to detected collinearity among covariates, our study did not account for numerous covariates, potentially introducing some degree of error into the results.** However, on the flip side, it is worth noting that this study may be less susceptible to confounding factors.

It is now evident that SAAC can effectively identify individuals with an elevated risk of all-cause mortality. The potential utility of this information extends to aiding in treatment decisions, fostering patients’ awareness of disease risks and symptoms, serving as a motivational tool for lifestyle decisions and changes, enhancing individual risk prediction, and presenting new targets for innovative treatments. Furthermore, future research should delve into whether knowledge about AAC has enhanced primary prevention and clinical management strategies. Given its potential to complement the assessment of coronary artery calcification, it holds promise for contributing to the early detection and primary prevention strategies of prevalent clinical cardiovascular diseases.

## Conclusions

In conclusion, our study, based on a nationally representative sample, establishes a significant association between AAC and an increased risk of all-cause mortality. The findings underscore the importance of considering AAC as a predictive marker for adverse health outcomes in the general population of the U.S. Further research and clinical attention to AAC could enhance risk prediction and inform preventive strategies.

## Supporting information

S1 Table Baseline characteristics of the included and excluded populations. (DOCX)

## Abbreviations

AAC: Abdominal aortic calcification
CVD: Cardiovascular Disease
HR: hazard ratio
MAAC: mild–moderate abdominal aortic calcification
NHANES: National Health and Nutrition Examination Survey
NCHS: National Center for Health Statistics
PIR: Poverty–income ratio
RCS: Restricted cubic spline
RR: risk ratio
SAAC: Severe abdominal aortic calcification
