# Supplementary material for: Association of the abdominal aortic calcification with all-cause and cardiovascular disease-specific mortality: Prospective cohort study
Source: PLoS One. 2025 Jan 16;20(1):e0314776. doi: 10.1371/journal.pone.0314776 (PMC11737679; doi:10.1371/journal.pone.0314776)
Supplement: S1 Table — (DOCX) [file pone.0314776.s001.docx]

**Supplementary Data**

Table S1. Baseline characteristics of the included and excluded populations.

| Characteristic | All (n = 146135121) ^a^ | Include (n = 99008875) | Exclude (n = 47126246) ^a^ | *P* Value |
| --- | --- | --- | --- | --- |
| Age, years | 57.90 (11.74) | 57.42 (11.53) | 59.45 (12.27) | <0.001 |
| Sex, % |  |  |  | 0.018 |
| Male | 69015087 (47.23%) | 54401503 (48.66%) | 14613583 (42.56%) |  |
| Female | 77120034 (52.77%) | 57397772 (51.34%) | 19722261 (57.44%) |  |
| Race, % |  |  |  | <0.001 |
| Mexican American | 10277946 (7.03%) | 7279573 (6.51%) | 2998372 (8.73%) |  |
| Other Hispanic | 6390826 (4.37%) | 4826861 (4.32%) | 1563965 (4.55%) |  |
| Non-Hispanic White | 103515423 (70.84%) | 80879994 (72.34%) | 22635428 (65.92%) |  |
| Non-Hispanic Black | 15623350 (10.69%) | 10770883 (9.63%) | 4852466 (14.13%) |  |
| Non-Hispanic Asian | 7273594 (4.98%) | 5625387 (5.03%) | 1648207 (4.80%) |  |
| Other Race | 3053982 (2.09%) | 2416577 (2.16%) | 637405 (1.86%) |  |
| PIR | 3.09 (1.64) | 3.17 (1.63) | 2.72 (1.65) | <0.001 |
| Marital status. % |  |  |  | 0.004 |
| Married/Living with partner | 97325931 (66.64%) | 76338200 (68.28%) | 20987731 (61.28%) |  |
| Single | 48720693 (33.36%) | 35461075 (31.72%) | 13259617 (38.72%) |  |
| Education level, % |  |  |  | 0.017 |
| High school degree/equivalency or less | 55259182 (37.83%) | 14652378 (42.76%) | 40606804 (36.32%) |  |
| Some college or associates degree | 44407041 (30.40%) | 10396861 (30.34%) | 34010179 (30.42%) |  |
| College Graduate or above | 46400771 (31.77%) | 9218476 (26.90%) | 37182294 (33.26%) |  |

^a^: Missing values for marital status (n = 88497), education level (n = 68127), PIR (n = 9356132).

Data are presented as mean (SD), n (%), and *P* value.

Analysis conducted: Wilcoxon rank-sum test for complex survey samples; chi-squared test with Rao & Scott's second-order correction.

Abbreviations: PIR = poverty income ratio.
